# Initial drainage‐related prognostic factors for perihilar cholangiocarcinoma: A single‐center retrospective study

**DOI:** 10.1002/deo2.127

**Published:** 2022-05-22

**Authors:** Katsuhiko Sato, Minoru Shigekawa, Kazuhiro Kozumi, Junya Okabe, Yu Sato, Takeshi Tamura, Teppei Yoshioka, Ryotaro Sakamori, Yoshifumi Iwagami, Daisaku Yamada, Yoshito Tomimaru, Takehiro Noda, Hidenori Takahashi, Shogo Kobayashi, Hidetoshi Eguchi, Tomohide Tatsumi, Tetsuo Takehara

**Affiliations:** ^1^ Department of Gastroenterology and Hepatology Osaka University Graduate School of Medicine Osaka Japan; ^2^ Department of Gastroenterological Surgery Osaka University Graduate School of Medicine Osaka Japan

**Keywords:** cholangiocarcinoma, cholangitis, drainage, endoscopic retrograde cholangiopancreatography, endoscopic sphincterotomy

## Abstract

**Objectives:**

Perihilar cholangiocarcinoma (PCC) is a complex disorder involving the hepatic hilum. Multiple endoscopic retrograde cholangiopancreatography sessions are necessary for diagnosis and treatment with underlying cholangitis risk. Our aim is to clarify the initial‐drainage‐related prognostic factors of PCC.

**Methods:**

This study was a single‐center retrospective study. A total of 104 consecutive patients diagnosed with PCC from January 2010 to February 2020 were enrolled. We defined the diagnostic period as the time between the first biliary drainage attempt and the final drainage when treatment, including surgery or chemotherapy, was started. We focused on this initial period and analyzed the endoscopy‐related factors that affected mortality.

**Results:**

Overall survival of all PCC patients was 599 days. Overall survival of surgically treated patients and unresectable patients were 893 days and 512 days, respectively. In 48 surgically treated patients, drainage‐related cholangitis within the diagnostic period, defined as new cholangitis that occurred after the first biliary drainage attempt, worsened overall survival from 1460 days to 607 days. Endoscopic sphincterotomy, the first drainage method other than endoscopic nasobiliary drainage, and four or more endoscopic retrograde cholangiopancreatography sessions were risk factors for drainage‐related cholangitis. Drainage‐related cholangitis increased pathological lymph node metastasis. Percutaneous transhepatic biliary drainage as final drainage was the only prognostic factor in unresectable chemotherapy‐treated patients.

**Conclusions:**

Drainage‐related cholangitis worsened the prognosis in PCC patients who underwent surgery. Appropriate endoscopic retrograde cholangiopancreatography strategies, especially during the diagnostic period, are of great importance in PCC.

## INTRODUCTION

Perihilar cholangiocarcinoma (PCC), located at the hepatic hilum, is the most common type of biliary cancer.^1^ Choosing a suitable treatment strategy for PCC is a major priority due to its complex origin, where all hepatic ducts, arteries, and portal veins gather. Surgical resection is the only way to cure PCC, as in most solid tumors. However, PCC resection requires significant expertise and is associated with high mortality, resulting in a 5‐year survival rate below 50%.^2^, ^3^ R0 resection is one of the strongest prognostic factors for the surgical treatment of PCC. Clarifying the extent of biliary invasion in individual hepatic ducts is essential for determining the possibility of surgical R0 resection.^4^ The Bismuth classification, focusing on secondary branch invasion, is often used to classify the extent of biliary invasion in PCC.^5^ Endoscopic retrograde cholangiopancreatography (ERCP) plays a significant role in clarifying the extent of invasion both by precise imaging of the entire intra‐ and extrahepatic bile duct and by pathological diagnosis via step biopsy. The indication for surgical resection differs between hospitals, and appropriate ERCP strategies are based on each center's criteria.

Biliary obstruction and jaundice are common characteristics associated with PCC. Preoperative biliary drainage of the future remnant liver improves liver function and enhances regenerative capacities of the liver. Incomplete drainage of the remnant liver is a mortality risk when the future remnant liver volume is below 50%.^6^ In addition, selective drainage of infected segments of the liver decreases morbidity after hepatectomy.^7^ A meta‐analysis supporting preoperative drainage has been reported, suggesting that preoperative drainage may decrease postoperative morbidity.^8^ In contrast, biliary drainage has been repeatedly shown to increase postoperative infection‐related adverse events.^9^, ^10^ Cholangitis has been observed in 68% of patients with biliary drainage and 0% of patients without preoperative drainage.^6^ In a meta‐analysis focusing on the effect of preoperative cholangitis on PCC, preoperative cholangitis was associated with postoperative mortality, morbidity, risk of liver failure, and infection.^11^ There are pros and cons of preoperative biliary drainage, as shown above; however, in Japanese clinical practice, preoperative biliary drainage is widely performed and expected to benefit patients with hepatectomy.^12^

The aim of our study was to clarify the drainage‐related prognostic factors within the diagnostic period from the viewpoints of endoscopists. Biliary drainage and diagnostic ERCP are essential features of PCC diagnosis and treatment. Previous reports concerning PCC have focused mainly on surgical resection and presurgical factors, and few reports have emphasized the importance of factors associated with ERCP. Repeated and inappropriate drainage may trigger cholangitis, which increases postoperative morbidity and interrupts chemotherapy. Identifying the drainage‐related risks associated with PCC will help those performing ERCP determine what should be done and what must be avoided in the primary drainage and diagnosis of suspected PCC.

## METHODS

### Patients

Consecutive patients diagnosed with or treated for PCC at Osaka University Hospital from January 2010 to February 2020 were included. All patients experienced prior expert conferences, and PCC was described as the major prognostic factor. The patients eligible for the study were identified from our hospital databases and were retrospectively reviewed. This study adhered to the Declaration of Helsinki and was approved by the Institutional Review Board of Osaka University (Approval Number: 16318).

### Definitions

Patients with a pathological diagnosis of or presumed PCC according to expert conferences were diagnosed with PCC. We defined the first biliary drainage attempt performed against suspected PCCs as the first drainage. Details of the final drainage are described below. In surgically treated patients, drainage just before hepatectomy, preoperative hospital discharge, or successful neoadjuvant chemotherapy induction represented the final drainage. In unresectable patients receiving chemotherapy, biliary drainage in which chemotherapy could be started and that was associated with hospital discharge was referred to as final biliary drainage. In patients receiving best supportive care, biliary drainage performed just before the best supportive care decision was used as the final drainage. For our investigation of diagnostic ERCP and biliary drainage performed primarily on suspected PCCs, we defined the period between the date of the first drainage and final drainage as the diagnostic period (Figure S1). Endoscopists with experience of 6–8 years started every ERCP session, and experts took cover according to the difficulty of the examination.

Cholangitis was diagnosed according to the Tokyo Guidelines 2018.^13^ Prediagnostic cholangitis was defined as cholangitis before the first drainage, including at the time of the first visit to the hospital. Drainage‐related cholangitis was defined as new cholangitis that emerged after the first biliary drainage attempt within the diagnostic period. In patients with prediagnostic cholangitis, new cholangitis that occurred after prediagnostic cholangitis had resolved was considered drainage‐related cholangitis.

Successful biliary drainage was defined as a decrease in the total bilirubin level by 50% or reaching the normal limit within 14 days.^14^ To simplify the drainage area, we divided the liver into three segments, namely, the left lobe, anterior segment, and posterior segment, and addressed each segment as being of equal value within this study. Post‐ERCP pancreatitis was defined as an amylase level three times the normal limit 24 h after ERCP and concomitant abdominal pain. Severity was scored according to Cotton's criteria.^15^

Overall survival (OS) was the time from the first visit to the hospital with assumed PCC to the date of death regardless of the cause or the last date of the hospital visit. Time to diagnosis was the time from the first visit to the hospital with suspected PCC to the date of the first pathological diagnosis. For staging, the criteria of the 6th version of the general rules for clinical and pathological studies on cancer of the bile duct published by the Japanese Society of Hepatobiliary‐Pancreatic Surgery were used. Staging and Bismuth classification were identified clinically with contrast‐enhanced multidetector computed tomography/magnetic resonance imaging before drainage or ERCP. Clinical lymph node metastasis was defined as fluorine‐18 deoxyglucose accumulation with positron emission tomography‐computed tomography (CT) or a minor diameter over 10 mm based on CT.

### Outcome measurement

The primary endpoint of this study was to clarify the drainage‐related prognostic factors within the diagnostic period. We focused mainly on the factors related to diagnosis and biliary drainage from the viewpoints of endoscopists. The variables associated with patient characteristics and ERCP procedures were retrospectively collected based on medical records.

### Statistical analysis

The data are shown as median values with the range or IQR unless otherwise specified. For the univariate analysis, the Mann‐Whitney U test was used for continuous variables, the Wilcoxon rank‐sum test was used for ordinal scale variables, and the chi‐square test was used for categorical variables. A Cox proportional hazard model was used for univariate and multivariate analyses of prognostic factors. A logistic regression model was used for multivariate analysis to analyze the risk of related factors. For the multivariate analysis, factors shown to be significant in the univariate analysis were applied. OS was estimated with the Kaplan–Meier method and statistically evaluated by the log‐rank test. A *p*‐value below 0.05 was considered significant. All statistical analyses were performed with JMP Pro 15 (SAS Institute Inc., Tokyo, Japan).

## RESULTS

### Patients

A total of 119 patients were presumed to have PCC from January 2010 to February 2020 at Osaka University Hospital (Figure S2). Fifteen patients were excluded for the following reasons: nine with a second opinion at our hospital; four referred but returned to the original hospital without any treatment; one with recurrent PCC primarily treated at a different hospital; and one who was referred for radiotherapy and continued treatment at the original hospital. Of the 104 patients analyzed, 48 underwent surgical resection. Of the 56 unresectable patients, 44 underwent chemotherapy. Patient baseline characteristics are shown (Table 1). There were 68 male and 36 female patients. The median age was 71 years. Compared with surgically treated patients, unresectable patients had a shorter follow‐up period with a median of 406 days (vs. 632 days), an advanced clinical stage, advanced tumor factor, advanced metastasis factor, more prediagnostic cholangitis, a longer diagnostic period, more frequent ERCP/percutaneous transhepatic biliary drainage (PTBD) sessions within the diagnostic period, and more drainage‐related cholangitis (Table 1).

**TABLE 1:** Patient baseline characteristics and univariate analysis

Variable		All patients (*n* = 104)	Surgically treated (*n* = 48)	Unresectable (*n* = 56)	*p*‐value
Sex	Male/female	68/36	33/15	35/21	0.504
Age, years	Median (range)	71 (36–86)	71 (36–79)	72 (36–86)	0.825
ASA PS, class	1/2/3	52/33/19	22/19/7	30/14/12	0.259
Time to diagnosis, days	Median (IQR)	32 (11–65)	36 (11–69)	30 (11.5–60)	0.724
Follow‐up period, days	Median (IQR)	509 (287–824)	632 (444–1025)	406 (137–572.5)	<0.0001
Bismuth‐Corlette type	I/II/IIIa/IIIb/IV	15/11/44/15/19	3/8/19/10/8	12/3/25/5/11	0.317
Clinical stage	I/II/IIIa/IIIb/IVa/IVb	1/11/21/15/25/31	1/8/13/12/14/0	0/3/8/3/11/31	<0.0001
Clinical T	T1a/T1b/T2a/T2b/T3/ T4a/T4bT1a/T1b/T2a/T2b/T3/ T4a/T4b	0/1/10/10/42/9/32	0/1/6/7/21/3/10	0/0/4/3/21/6/22	0.009
Clinical N	N0/N1	65/40	16/32	23/33	0.415
Clinical M	M0/M1	74/30	48/0	26/30	<0.0001
AST, IU/L	Median (range)	95 (18–969)	112 (22–969)	83 (18–674)	0.487
ALT, IU/L	Median (range)	156 (7–1080)	177 (15–766)	122 (7–1080)	0.249
ALP, IU/L	Median (range)	972 (277–4485)	998 (308–2717)	841 (277–4485)	0.469
GGT, IU/L	Median (range)	684 (20–3473)	724 (136–1962)	594 (20–3473)	0.157
T‐Bil, mg/dl	Median (range)	4.8 (0.3–25.2)	2.70 (0.5–21.5)	5.68 (0.3–25.2)	0.184
Prediagnostic cholangitis	Yes/no	31/73	8/40	23/33	0.007
First drainage method^†^	ENBD/PS/inside stent/PTBD/ incomplete study/no data	61/31/2/3/6/1	30/9/1/3/4/1	31/22/1/0/2/0	0.088
First drainage segments	0/1/2/3/no data	6/86/11/0/1	4/39/4/0/1	2/47/7/0	0.572
EST (including previous)	Yes/no	41/63	18/30	23/33	0.710
Successful biliary drainage (within 2 weeks)	Yes/no/no data	74/27/3	36/10/2	38/17/1	0.300
Successful biliary drainage (no time limit)	Yes/no	103/1	48/0	55/1	0.352
Diagnostic period, days	Median (IQR)	23 (8–55)	17 (6–32)	33 (14–67)	0.007
No of ERCP/PTBD within diagnostic period	Median( range)	3 (1–13)	2.5 (1–13)	3 (1–12)	0.031
Drainage‐related cholangitis	Yes/no	52/52	19/29	33/23	0.049
Final drainage method^†^	ENBD/PS/inside stent/EMS/PTBD/ no stent	4/58/17/6/12/7	3/22/12/0/7/4	1/36/5/6/5/3	0.022
Final drainage segments	0/1/2/3	7/65/28/4	4/32/11/1	3/33/17/3	0.196
Post‐ERCP pancreatitis	No pancreatitis/mild/moderate/severe/no data	86/1/11/4/2	41/0/4/3/0	45/1/7/1/2	0.627

ASA‐PS, American Society of Anesthesiologists physical status; IQR, interquartile range; AST, aspartate transaminase; ALT, alanine transaminase; ALP, alkaline phosphatase; GGT, gamma‐glutamyltransferase; T‐Bil, total bilirubin; ENBD, endoscopic nasobiliary drainage; PS, plastic stent deployed transpapillary; PTBD, percutaneous transhepatic biliary drainage; EST, endoscopic sphincterotomy; ERCP, endoscopic retrograde cholangiopancreatography; No., number; EMS, expandable metallic stent.

ΠComparisons of surgical patients and unresectable patients are shown as *p*‐values.

^†^ ENBD+X→ENBD, PTBD+X→PTBD, PS+inside→PS

### Prognostic factors for all 104 PCC patients

**FIGURE 1:**
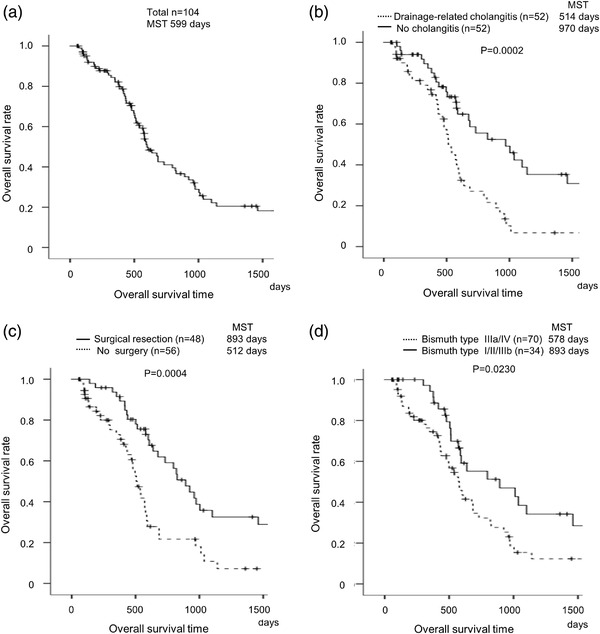
**Overall survival of patients with perihilar cholangiocarcinoma**. Overall survival (OS) of 104 perihilar cholangiocarcinoma patients based on the Kaplan‐Meier method. (a) Median survival time (MST) was 599 days. (b) Patients with drainage‐related cholangitis had a shorter OS than those without cholangitis (MST: 514 days vs. 970 days, *p* = 0.0002, log‐rank test). (c) Patients who underwent surgical resection had a longer OS than those who did not (MST: 893 days vs. 512 days, *p* = 0.0004, log‐rank test). (d) Patients with Bismuth type IIIa/IV had a shorter OS than patients with Bismuth type I/II/IIIb (MST: 578 days vs. 893 days, *p* = 0.0230, log‐rank test)

The median survival time (MST) of the 104 PCC patients was 599 days (Figure 1a). Univariate analysis indicated that the factors associated with OS in all 104 PCC patients were surgical resection, Bismuth type, endoscopic sphincterotomy (EST), drainage‐related cholangitis, and total bilirubin level (Table 2). In the multivariate analysis of these factors, surgical resection, Bismuth type, and drainage‐related cholangitis were significant factors associated with OS (Table 2). Drainage‐related cholangitis shortened OS, while surgical resection and Bismuth type I/II/IIIb improved survival (Figure 1b–d).

**TABLE 2:** Prognostic factors for all perihilar cholangiocarcinoma patients

			Univariate analysis			**Multivariate analysis**		
Variable		***n***	**HR**	**95% CI**	***p*‐value**	**HR**	**95% CI**	***p*‐value**
**Sex**	Male	68	0.79	0.473–1.334	0.384			
	Female	36	1					
**Age**	≥75years	41	1.2	0.733–1.974	0.465			
	<75years	63	1					
**Surgical resection**	Yes	48	0.41	0.244–0.680	0.001	0.44	0.253–0.772	0.004
	No	56	1			1		
**ASA‐PS**	Class 2–3	51	0.79	0.485–1.302	0.362			
	Class 0–1	53	1					
**Bismuth type**	IIIa+IV	42	1.80	1.077–3.025	0.025	1.86	1.091–3.174	0.023
	I+II+IIIb	62	1			1		
**EST (including previous)**	Yes	41	1.68	0.359–0.986	0.044	1.61	0.901–2.894	0.108
	No	63	1			1		
**Prediagnostic cholangitis**	Yes	31	0.96	0.564–1.649	0.895			
	No	73	1					
**Drainage‐related cholangitis**	Yes	52	2.61	1.556–4.385	0.0003	2.44	1.416–4.215	0.001
	No	52	1			1		
**First drainage segments** **^#^**	2–3 segments	11	1,47	0.629–3.455	0.371			
	1 segment	86	1					
**Final drainage segments** **^#^**	2–3 segments	32	1.43	0.844–2.407	0.185			
	1 segment	65	1					
**Type of first drainage** **^#^**	ENBD	61	0.78	0.463–1.330	0.368			
	Not ENBD	36	1					
**No. of ERCP/PTBD sessions within diagnostic period**	4 times or more	32	1.61	0.953–2.711	0.075			
	1–3 times	72	1					
**PTBD as final drainage**	PTBD	12	1.86	0.942–3.659	0.074			
	Not PTBD	92	1					
**T‐Bil** **^#^**	>3 mg/dl	57	2.06	1.201–3.540	0.009	1.48	0.825–2.671	0.187
	<3 mg/dl	41	1			1		
**ALP** **^#^**	>1800 IU/L	28	0.69	0.366–1.308	0.257			
	<1800 IU/L	67	1					
**GGT** **^#^**	>200 IU/L	83	0.77	0.373–1.577	0.470			
	<200 IU/L	11	1					

HR, hazard ratio; CI, confidence interval; ASA‐PS, American Society of Anesthesiologists Physical Status; EST, endoscopic sphincterotomy; ENBD, endoscopic nasobiliary drainage; No., number; ERCP, endoscopic retrograde cholangiopancreatography; PTBD, percutaneous transhepatic biliary drainage; T‐Bil, total bilirubin; ALP, alkaline phosphatase; GGT, gamma‐glutamyltransferase.

^#^ Patients without data or stenting were excluded

### Surgically treated patients and prognostic factors

**FIGURE 2:**
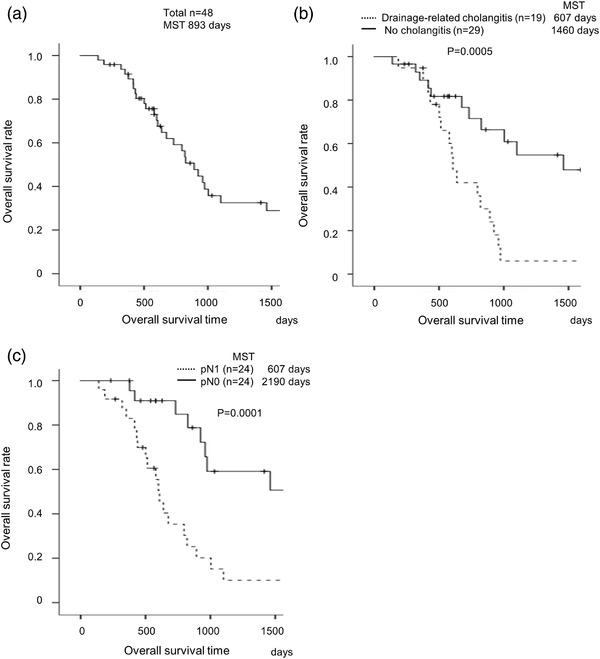
**Overall survival of surgically treated patients**. Overall survival as determined by the Kaplan‐Meier method of 48 surgically treated patients. (a) Median survival time (MST) was 893 days. (b) Patients with drainage‐related cholangitis had a shorter overall survival than those without cholangitis (MST: 607 days vs. 1460 days, *p* = 0.0005, log‐rank test). (c) Patients with pathological lymph node metastasis had a shorter overall survival than those without pathological lymph node metastasis (MST: 607 days vs. 2190 days, *p* = 0.0001, log‐rank test)

The MST of the 48 patients who underwent surgery was 893 days (Figures 1c and 2a). Univariate analysis indicated that the factors associated with OS were drainage‐related cholangitis and endoscopic nasobiliary drainage (ENBD) as the first drainage (Table 3). In the multivariate analysis, drainage‐related cholangitis was the only prognostic factor (HR 4.69, *p* = 0.003, Table 3). The MSTs of the drainage‐related cholangitis group and no cholangitis group were 607 and 1460 days, respectively (*p* = 0.0005, Figure 2b). The risk factors for drainage‐related cholangitis in the surgically treated patients were analyzed with a logistic regression model. Univariate analysis showed EST, ENBD as the first drainage, and four or more ERCP sessions/PTBDs within the diagnostic period as factors related to drainage‐related cholangitis. In the multivariate analysis, EST (odds ratio [OR] 5.97, *p* = 0.042), ENBD as first drainage (OR 0.17, *p* = 0.047), and four or more ERCP/PTBD sessions within the diagnostic period (OR 36.30, *p* = 0.007) were independent factors of drainage‐related cholangitis (Table 4).

**TABLE 3:** Prognostic factors for surgically treated perihilar cholangiocarcinoma patients

			**Univariate analysis**			**Multivariate analysis**		
**Variable**		***n***	**HR**	**95% CI**	***p*‐value**	**HR**	**95% CI**	***p*‐value**
Sex	Male	33	1.15	0.502–2.622	0.746			
	Female	15	1					
Age	≥75 years	20	1.42	0.685–2.962	0.344			
	<75 years	28	1					
Preoperative chemotherapy	Yes	7	1.64	0.563–4.761	0.365			
	No	41	1					
ASA‐PS	Class 2–3	26	0.87	0.419–1.826	0.721			
	Class 0–1	22	1					
T‐Bil^#^	≥3 mg/dl	22	2.05	0.938–4.498	0.072			
	<3 mg/dl	23	1					
Bismuth type	IIIa+IV	27	1.75	0.825–3.709	0.144			
	I+II+IIIb	21	1					
EST(including previous)	Yes	18	1.99	0.912–4.333	0.084			
	No	30	1					
Pre‐diagnostic cholangitis	Yes	8	0.53	0.201–1.410	0.205			
	No	40	1					
Drainage‐related cholangitis	Yes	19	3.78	1.711–8.340	0.001	4.69	1.671–13.142	0.003
	No	29	1			1		
First drainage segments^#^	2–3 segments	4	2.00	0.441–9.044	0.370			
	1 segment	39	1					
Final drainage segments^#^	2–3 segments	12	1.86	0.823–4.203	0.136			
	1 segment	32	1					
Type of first drainage^#^	ENBD	30	0.32	0.134–0.746	0.009	0.51	0.203–1.289	0.155
	Not ENBD	13	1			1		
No. of ERCP/PTBD sessions within diagnostic period	four times or more	9	2.19	0.886–5.398	0.090			
	1–3 times	39	1					
PTBD as final drainage	PTBD	7	1.86	0.751–4.604	0.180			
	Not PTBD	41	1					

HR, hazard ratio; CI, confidence interval; ASA‐PS, American Society of Anesthesiologists Physical Status; T‐Bil, total bilirubin; EST, endoscopic sphincterotomy; ENBD, endoscopic nasobiliary drainage; No., Number; ERCP, endoscopic retrograde cholangiopancreatography; PTBD, percutaneous transhepatic biliary drainage. ^#^Patients without data or with no stents were excluded.

**TABLE 4:** Risk factors for drainage‐related cholangitis in surgically treated perihilar cholangiocarcinoma patients

			**Univariate analysis**			**Multivariate analysis**		
**Variable**		***n***	**OR**	**95% CI**	***p*‐value**	**OR**	**95% CI**	***p*‐value**
Sex	Male	33	0.98	0.280–3.392	0.968			
	Female	15	1					
Age	≥75 years	20	1.03	0.319–3.329	0.960			
	<75 years	28	1					
Preoperative Chemotherapy	Yes	7	0.43	0.085–2.200	0.313			
	No	41	1					
ASA‐PS	Class 2–3	26	2.67	0.793–8.969	0.113			
	Class 0–1	22	1					
T‐Bil^#^	≥3 mg/dl	22	1.90	0.155–1.781	0.301			
	<3 mg/dl	23	1					
Bismuth type	IIIa+IV	27	2.32	0.692–7.792	0.173			
	I+II+IIIb	21	1					
EST(including previous)	Yes	18	4.32	1.243–15.024	0.021	5.97	1.070–33.377	0.042
	No	30	1			1		
Prediagnostic cholangitis	Yes	8	1.67	0.362–7.671	0.512			
	No	40	1					
First drainage segments^#^	2–3 segments	4	1.60	0.203–12.596	0.655			
	1 segment	39	1					
Type of first drainage^#^	ENBD	30	0.16	0.039–0.675	0.012	0.17	0.0280–0.979	0.047
	Not ENBD	13	1			1		
No. of ERCP/PTBD sessions within diagnostic period	4 times or more	9	7.88	1.421–43.630	0.018	36.30	2.737–482.024	0.007
	1–3 times	39	1			1		
			

OR, odds ratio; CI, confidence interval; ASA‐PS, American Society of Anesthesiologists Physical Status; T‐Bil, total bilirubin; EST, endoscopic sphincterotomy; ENBD, endoscopic nasobiliary drainage; No., number; ERCP, endoscopic retrograde cholangiopancreatography; PTBD, percutaneous transhepatic biliary drainage. **^#^** Patients without data or with no stents were excluded

Regarding the operative findings, no difference was observed between the drainage‐related cholangitis group and the no cholangitis group with respect to R0 resection, preoperative or postoperative chemotherapy, lymphatic vessel invasion, vascular invasion, and neural invasion (Table 5). The percentage of overall recurrence did not differ between the drainage‐related cholangitis group and the no cholangitis group. Changes in pathological lymph node metastasis were the only pathological factor related to prognosis observed in the drainage‐related cholangitis group compared with the no cholangitis group (Table 5 and Figure 2c).

**TABLE 5:** Pathological features of patients with or without drainage‐related cholangitis

		**Drainage‐related cholangitis**		
**Variable**		**Yes (*n* = 19)**	**No (*n* = 29)**	***p*‐value**
Preoperative chemotherapy^$^	Yes/no	4 (1)/15	3 (2)/26	0.304
R0 resection	Yes/no	17/2	27/2	0.656
Histology	Pap/tub/por	0/19/0	2/23/4	0.106
Stroma	Med/int/sci	0/17/2	1/20/8	0.236
Invasion type	INFa/INFb/INFc	0/17/3	1/18/10	0.214
Lymphatic vessel invasion	ly0/ly1/ly2	10/8/1	12/16/1	0.519
Vascular invasion	v0/v1/v2	13/6/0	19/8/2	0.720
Neural invasion	ne0/ne1/ne2/ne3	2/3/7/7	3/6/11/9	0.682
pT	pT1a/pT2a/pT2b/pT3/pT4a/pT4b	0/1/3/10/3/0/3	0/1/7/15/6/0/0	0.217
pN	pN0/pN1	5/14	19/10	0.008
Adjuvant chemotherapy	Yes/no	3/16	5/24	0.895
Postoperative recurrence^#^	Yes/no	12/5	15/12	0.319

pap, papillary adenocarcinoma; tub, tubular adenocarcinoma; por, poorly differentiated adenocarcinoma; med, medullary type; int, intermediate type; sci, scirrhous type; INF, infiltration

^$^ patients with neoadjuvant chemotherapy are shown in parenthesis.

^#^ patients without R0 resection are excluded.

### Unresectable patients and prognostic factors

The MST of all 56 unresectable patients was 512 days (Figure 1c and Figure S3a). Among the unresectable patients, chemotherapy and PTBD as final drainage were the prognostic factors upon univariate and multivariate analysis (Table S1). The MST of patients with chemotherapy was 536 days, and that of patients who did not receive chemotherapy was 204 days (*p* = 0.009, Figure S3b). The MST of patients without PTBD was 536 days, and that of patients with PTBD was 209 days (*p* = 0.0005, Figure S3c). We performed further analysis of unresectable patients receiving chemotherapy. Of the 44 patients who received chemotherapy, 41 patients who were treated at our institute were analyzed. PTBD as final drainage was the only prognostic factor in unresectable chemotherapy‐treated patients (Table S2 and Figure S3d).

## DISCUSSION

ERCP plays an important role in patients with suspected PCC. Our study focuses on the period of primary drainage and diagnosis of suspected PCCs and defines this time as the diagnostic period. During this period, we often experience a delay in treatment due to difficulties associated with biliary drainage and unclear histopathological diagnosis. Multiple sessions of ERCP frequently cause drainage‐related cholangitis, resulting in re‐drainage. If drainage‐related cholangitis can be avoided, biliary drainage must have a stronger positive influence on the prognosis of PCC patients. Indeed, in our study, drainage‐related cholangitis was a prognostic factor associated with OS in preoperative patients and seemed to be the main prognostic factor within the diagnostic period. However, drainage‐related cholangitis did not affect OS in unresectable and chemotherapy‐treated patients. Most patients with chemotherapy suffer from repeated cholangitis in the overall treatment period, resulting in a decrease in the direct impact of drainage‐related cholangitis within the initial diagnostic period. No endoscopic prognostic factor within the diagnostic period could be detected in chemotherapy‐treated patients in our study, and from this point of view, the diagnostic period is of stronger importance in surgically treated patients than in unresectable patients.

We have shown that EST, multiple drainages within the diagnostic period, and ENBD as the first drainage were factors associated with drainage‐related cholangitis in surgically treated patients. EST, the first factor detected in our study, and cholangitis are closely related. In patients with common bile duct stones, EST has been reported to cause acute cholangitis in 31% and liver abscess in 11% of patients.^16^ In this previous report, all cholangitis patients had residual intrahepatic stones, similar to cholangiocarcinoma in that cholestasis occurs in the intrahepatic ducts. EST did not decrease the frequency of post‐ERCP pancreatitis in PCC patients in our study (data not shown), and considering these results, EST without any specific reason must be avoided in the diagnostic period for suspected PCC patients. In our study, multiple drainage events within the diagnostic period were the second risk factor for drainage‐related cholangitis. ERCP itself and biliary stenting are known risk factors for cholangitis.^6^, ^17^ Multiple ERCP sessions provide multiple opportunities for enteral bacteria to enter the bile duct, which may increase the risk of cholangitis. The pre‐ERCP strategy of diagnosis and appropriate biliary drainage is important in reducing the risk of drainage‐related cholangitis. Next, ENBD as the first drainage was the third and only protective factor against drainage‐related cholangitis. The drainage method that is most beneficial to PCC patients has always been a question. ENBD is superior to endoscopic biliary stenting (EBS) in reducing the risk of obstructive cholangitis and stent dysfunction.^18^, ^19^ A previous study reported that the incidence of stent dysfunction 30 days after the initial drainage was 30% in the plastic stent group and 8% in the ENBD group.^19^ From our data, ENBD within the drainage period did not affect the mortality of all PCC patients (data not shown). However, we have shown that successful first drainage with ENBD reduced the risk of drainage‐related cholangitis in surgically treated PCC patients. In our institute, ENBD is the first choice for primary drainage in suspected PCC patients given the advantages of ENBD over EBS, such as repetitive bile cytology and cholangiography. After diagnosing the extent of biliary invasion and confirming which segment of the liver needs drainage to maintain liver function, we deploy mostly plastic inside stents in place of ENBD. ENBD as the first drainage method may benefit prediagnosed PCC patients with resectability.

We investigated why drainage‐related segmental cholangitis is associated with worse survival in surgically treated patients. When we compared the drainage‐related cholangitis group with the no cholangitis group, more pathological lymph node metastasis was observed in the cholangitis group. Pathological lymph node metastasis is one of the strongest prognostic factors for PCC. The 5‐year survival rate associated with PCC with pN1 is 20%, while that of PCC with pN0 is 80%.^20^ Inappropriate cholangiography of the undrained areas may trigger drainage‐related cholangitis in these areas. The increase in intrabiliary pressure and vascular permeability due to segmental cholangitis in areas with PCC may promote local lymph node metastasis. As in many cancers, inflammation of the bile duct and carcinogenesis in cholangiocarcinoma are closely related. The known risk factors for cholangiocarcinoma are primary sclerosing cholangitis and intrahepatic biliary stones, which both involve chronic inflammation via cholestasis. Inflammation is reported to enhance the metastatic abilities of cancer cells by maintaining cancer stem cells.^21^ The proinflammatory cytokine TNF‐a triggers endothelial mesenchymal transition in cholangiocarcinoma, promoting a tendency toward metastasis.^22^ These basic data support the possibility that inflammation may shorten the OS of PCC patients by promoting lymph node metastasis.

Our study has limitations. First, our study is a single‐center retrospective study. A multicenter randomized study is required to show whether the identified factors, such as EST, are associated with worse survival via an increase in drainage‐related cholangitis. We also could not collect adequate information to identify the details of drainage‐related cholangitis because our study was retrospective. Therefore, important factors of drainage‐related cholangitis, including whether drainage‐related cholangitis occurred in drained or undrained areas and the reasons for drainage‐related cholangitis like inappropriate biliary drainage and stent dysfunction, could not be assessed. Second, our survival curve was generated using patient data within 5 years of surgical resection, making our survival data inaccurate. Therefore, we could not analyze recurrence‐free survival, an important factor in postoperative survival. Third, our stenting methods and types of stents used were not consistent during the study period. For this reason, we could not clarify which type of biliary stenting affects prognosis, even though biliary stenting is a major factor in biliary drainage in PCC.

Here, we found that drainage‐related cholangitis was a prognostic factor for surgically treated patients, and EST, multiple ERCP sessions, and ENBD as the first drainage were factors related to drainage‐related cholangitis. PCC requires a multiple biliary approach to clarify the possibility of surgical resection and sustain effective drainage. During the diagnostic period, we must consider that all patients without obvious distant metastasis are candidates for R0 resection. Avoiding drainage‐related cholangitis within the diagnostic period may improve the survival of PCC patients.

## CONFLICT OF INTEREST

The authors declare no conflict of interest.

## FUNDING INFORMATION

The authors received no financial support for this study.

## Supporting information


**Figure S1. Definitions of Diagnostic Period in Perihilar Cholangiocarcinoma**. In surgically treated patients, the diagnostic period was defined as the time from the first biliary drainage to the final drainage surgery that was performed, discharge from the hospital prior to surgery, or successful neoadjuvant therapy induction. In chemotherapy‐treated patients, the diagnostic period was defined as the time from the first biliary drainage to the final drainage, after which chemotherapy‐induced patients could be discharged from the hospital. In best supportive care patients, the diagnostic period was defined as the time from the first biliary drainage to the final drainage, after which the decision of best supportive care was made.

**Figure S2. Patients Enrolled as Having Perihilar Cholangiocarcinoma**. One hundred nineteen patients were presumed to have perihilar cholangiocarcinoma from January 2010 to February 2020 at Osaka University Hospital. A total of 104 patients were enrolled in this study, and 15 patients were excluded, namely, nine patients who received a second opinion, four patients who were referred but returned to the original hospital without treatment, one patient with recurrent perihilar cholangiocarcinoma who was primarily treated in a different hospital, and one patient who was referred only for radiotherapy and continued treatment at the original hospital.

**Figure S3. Overall Survival of Unresectable Patients**. The overall survival (OS) and chemotherapy treatment time of 56 unresectable patients and 44 patients who underwent chemotherapy were analyzed by the Kaplan‐Meier method. a) The median survival time (MST) of unresectable patients was 512 days. b) Unresectable patients who received chemotherapy had a longer OS than those who did not receive chemotherapy (MST: 536 days vs. 204 days, *p* = 0.0090, log‐rank test). c) Unresectable patients with Percutaneous Transhepatic Biliary Drainage (PTBD) had a shorter OS than those without PTBD (MST: 209 days vs. 536 days, *p* = 0.0005, log‐rank test). d) Chemotherapy patients with PTBD had a shorter OS than those without PTBD (MST: 209 days vs. 478.5 days, *p* = 0.0040, log‐rank test).

Table S1. Prognostic factors for unresectable perihilar cholangiocarcinoma patients

Table S2. Prognostic factors for unresectable chemotherapy‐treated patients
