# Supplementary figures and images for: Initial drainage‐related prognostic factors for perihilar cholangiocarcinoma: A single‐center retrospective study
Source: DEN Open. 2022 May 22;3(1):e127. doi: 10.1002/deo2.127 (PMC9307739; doi:10.1002/deo2.127)

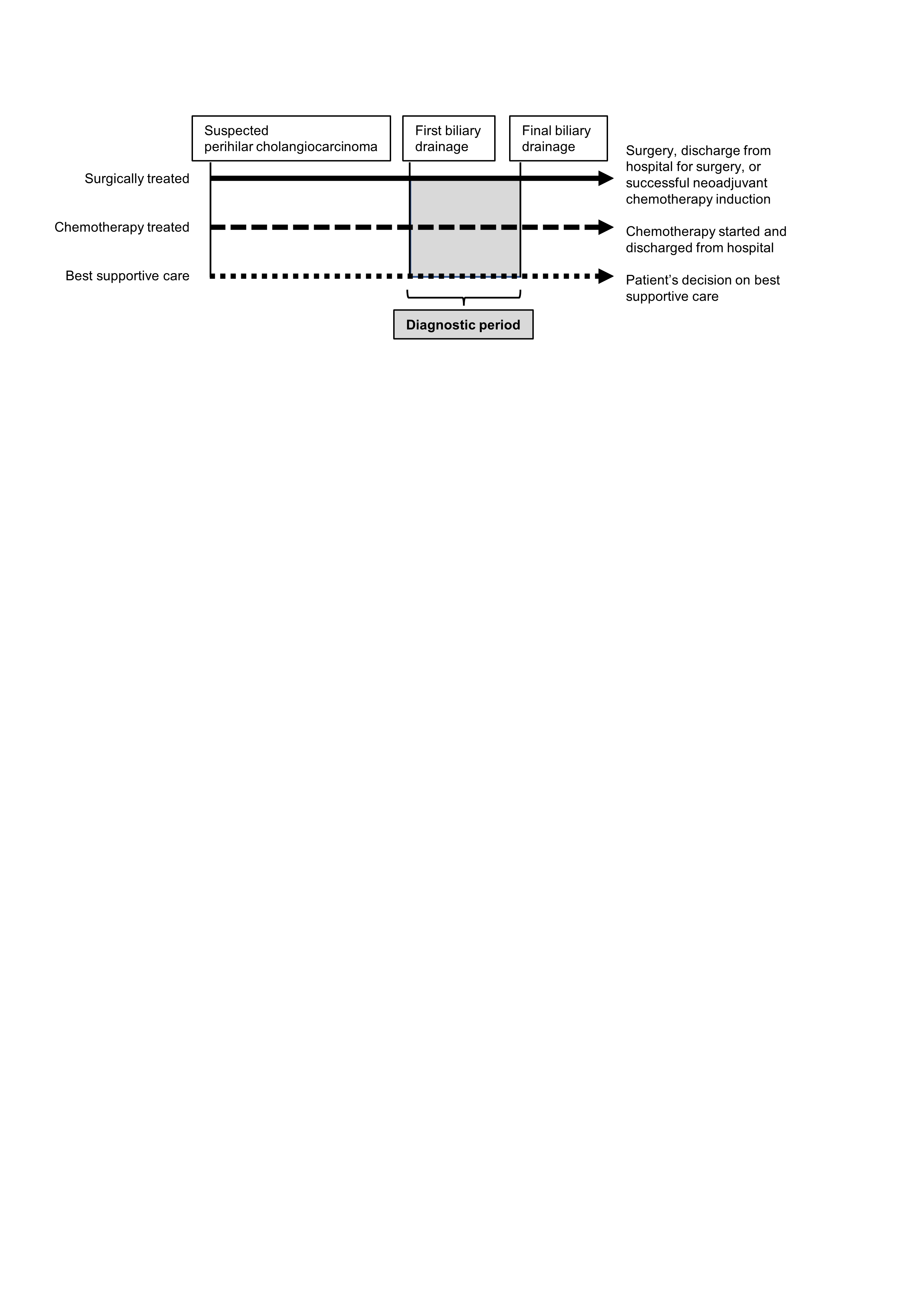

Supplement: Supplementary file 1 — Figure S1. Definitions of Diagnostic Period in Perihilar Cholangiocarcinoma. In surgically treated patients, the diagnostic period was defined as the time from the first biliary drainage to the final drainage surgery that was performed, discharge from the hospital prior to surgery, or successful neoadjuvant therapy induction. In chemotherapy‐treated patients, the diagnostic period was defined as the time from the first biliary drainage to the final drainage, after which chemotherapy‐induced patients could be discharged from the hospital. In best supportive care patients, the diagnostic period was defined as the time from the first biliary drainage to the final drainage, after which the decision of best supportive care was made. [file DEO2-3-e127-s005.TIF]

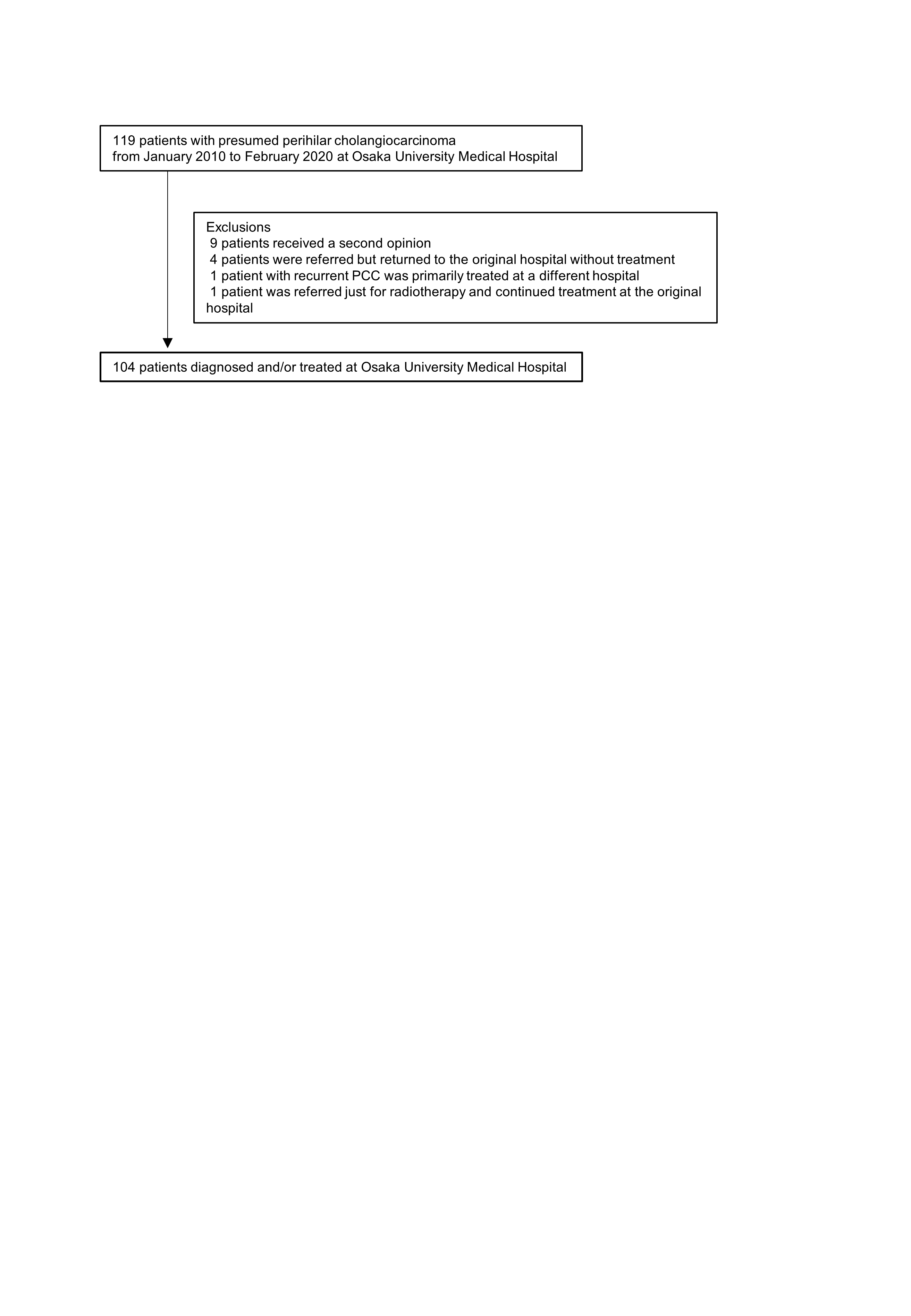

Supplement: Supplementary file 2 — Figure S2. Patients Enrolled as Having Perihilar Cholangiocarcinoma. One hundred nineteen patients were presumed to have perihilar cholangiocarcinoma from January 2010 to February 2020 at Osaka University Hospital. A total of 104 patients were enrolled in this study, and 15 patients were excluded, namely, nine patients who received a second opinion, four patients who were referred but returned to the original hospital without treatment, one patient with recurrent perihilar cholangiocarcinoma who was primarily treated in a different hospital, and one patient who was referred only for radiotherapy and continued treatment at the original hospital. [file DEO2-3-e127-s004.TIF]

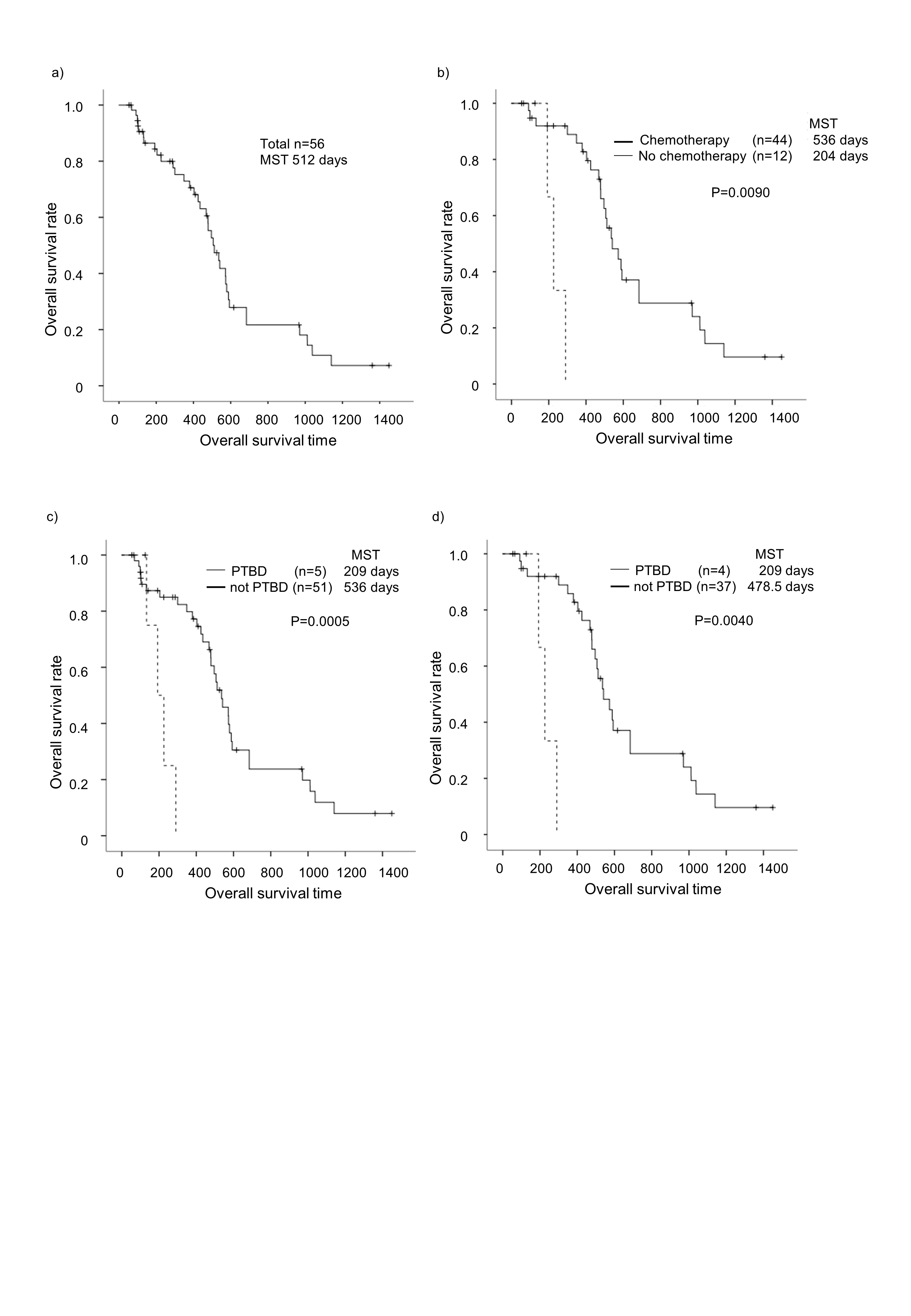

Supplement: Supplementary file 3 — Figure S3. Overall Survival of Unresectable Patients. The overall survival (OS) and chemotherapy treatment time of 56 unresectable patients and 44 patients who underwent chemotherapy were analyzed by the Kaplan‐Meier method. a) The median survival time (MST) of unresectable patients was 512 days. b) Unresectable patients who received chemotherapy had a longer OS than those who did not receive chemotherapy (MST: 536 days vs. 204 days, p = 0.0090, log‐rank test). c) Unresectable patients with Percutaneous Transhepatic Biliary Drainage (PTBD) had a shorter OS than those without PTBD (MST: 209 days vs. 536 days, p = 0.0005, log‐rank test). d) Chemotherapy patients with PTBD had a shorter OS than those without PTBD (MST: 209 days vs. 478.5 days, p = 0.0040, log‐rank test). [file DEO2-3-e127-s003.tif]
